# Effect of Asbestos Consumption on Malignant Pleural Mesothelioma in Italy: Forecasts of Mortality up to 2040

**DOI:** 10.3390/cancers13133338

**Published:** 2021-07-03

**Authors:** Enrico Oddone, Jordy Bollon, Consuelo Rubina Nava, Dario Consonni, Alessandro Marinaccio, Corrado Magnani, Antonio Gasparrini, Francesco Barone-Adesi

**Affiliations:** 1 Department of Public Health, Experimental and Forensic Medicine, University of Pavia, via Severino Boezio 24, 27100 Pavia, Italy; 2 Occupational Medicine Unit (UOOML), ICS Maugeri IRCCS, 27100 Pavia, Italy; 3 Department of Translational Medicine, University of Eastern Piedmont, 28100 Novara, Italy; jbollon94@gmail.com (J.B.); corrado.magnani@med.uniupo.it (C.M.); francesco.baroneadesi@uniupo.it (F.B.-A.); 4 Department of Economics and Statistics ‘Cognetti de Martiis’, University of Turin, 10124 Turin, Italy; consuelorubina.nava@unito.it; 5 Epidemiology Unit, Fondazione IRCCS Ca’ Granda Ospedale Maggiore Policlinico, 20122 Milan, Italy; dario.consonni@unimi.it; 6 Occupational and Environmental Medicine, Epidemiology and Hygiene Department, Italian Workers’ Compensation Authority (INAIL), 00187 Rome, Italy; a.marinaccio@inail.it; 7 Department of Public Health Environments and Society, London School of Hygiene & Tropical Medicine, London WC1H 9SH, UK; Antonio.Gasparrini@lshtm.ac.uk; 8 Centre for Statistical Methodology, London School of Hygiene & Tropical Medicine, London WC1E 7HT, UK

**Keywords:** asbestos consumption, forecasts, pleural mesothelioma, distributed lag non-linear model (DLNM), occupational medicine, epidemiology

## Abstract

**Simple Summary:**

Asbestos consumption figures could be used to construct epidemiological models to forecast the pleural mesothelioma mortality, although they are not frequently taken into consideration in this kind of analysis. Our purpose is to predict the future burden of pleural mesothelioma deaths in Italy until 2039 and to describe the exposure–response curve considering time lasted from the exposure start. Our results could be of interest to plan future needs of the Italian health system and to provide evidence on the consequences of asbestos exposures useful for countries in which this material is, at present, still used for productive activities.

**Abstract:**

Statistical models used to forecast malignant pleural mesothelioma (MPM) trends often do not take into account historical asbestos consumption, possibly resulting in less accurate predictions of the future MPM death toll. We used the distributed lag non-linear model (DLNM) approach to predict future MPM cases in Italy until 2040, based on past asbestos consumption figures. Analyses were conducted using data on male MPM deaths (1970–2014) and annual asbestos consumption using data on domestic production, importation, and exportation. According to our model, the peak of MPM deaths is expected to occur in 2021 (1122 expected cases), with a subsequent decrease in mortality (344 MPM deaths in 2039). The exposure–response curve shows that relative risk (RR) of MPM increased almost linearly for lower levels of exposure but flattened at higher levels. The lag-specific RR grew until 30 years since exposure and decreased thereafter, suggesting that the most relevant contributions to the risk come from exposures which occurred 20–40 years before death. Our results show that the Italian MPM epidemic is approaching its peak and underline that the association between temporal trends of MPM and time since exposure to asbestos is not monotonic, suggesting a lesser role of remote exposures in the development of MPM than previously assumed.

## 1. Introduction

Asbestos exposure, both in occupational and environmental settings, is estimated to annually cause around 255,000 deaths worldwide and an impressive amount in social costs and human suffering [1,2,3]. A large proportion of such deaths are due to Malignant Pleural Mesothelioma (MPM), a type of cancer that is almost exclusively caused by asbestos exposure [4]. Occupational exposure is considered the main determinant of the occurrence of MPM in men, while in women, para-occupational or environmental exposures play an important role, as well [5,6].

Italy was one of the most important producers and users of asbestos worldwide. National consumption of asbestos exceeded 100,000 tons in 1970 and further increased in the following years to reach 180,000 tons in 1980 [7,8]. Its largest use was in asbestos-cement production, followed by fire-proofing and thermal insulation in shipbuilding and railway carriages [9]. In 1992, a national ban forbade asbestos production, import, export, and trading in Italy. However, asbestos already in place has been only partially removed since then [10]. It has been calculated that at the beginning of the 2000s, there were still more than 70,000 workers in Italy that were exposed to asbestos, mainly in the construction and in the asbestos-removal sectors [11].

As the risk of MPM increases decades after asbestos exposure, many countries that banned asbestos at the end of the 20th century are just now approaching the peak of cases [12,13,14]. There is substantial uncertainty on how long the MPM epidemic will last both worldwide and in the countries that have banned asbestos use [15]. For this reason, up-to-date predictions of the future trend of MPM are necessary to inform public health interventions [12]. Several different approaches have been used to carry out such forecasts, such as age–cohort or age–period models [16,17], or age–period–cohort models (APC) [18]. However, these models do not take into account historical asbestos consumption, at the national or regional level, possibly resulting in less accurate predictions of the future MPM death toll. Therefore, models explicitly considering historical asbestos exposure (HAE) have been proposed and applied in different countries [19,20,21,22]. A possible limitation of currently used HAE models is that they use a fixed time-lag to predict the number of MPM cases according to the amount of asbestos consumption that occurred during a specific time in the past (e.g., 40 years before). However, it is plausible to expect more complex temporal patterns related to past asbestos consumption that results in biased projections of future trends of MPM. Distributed Lag Non-Linear Models (DLNMs) offer a flexible alternative to thoroughly describe associations in which the association between an exposure and an outcome is defined using flexible functions to account for potentially complex temporal dependencies [23]. While in recent years the use of DLNM has become relatively common to evaluate the health effects of environmental determinants such as air pollution and temperature, its application in other areas of epidemiology is still limited. In the present study, we used the DLNM approach to predict future MPM cases in Italy until 2040, based on past asbestos consumption figures after 1946.

## 2. Materials and Methods

### 2.1. Data Source

Analyses were conducted using data from the Italian National Statistical Institute (ISTAT) on male MPM deaths that occurred in the period 1970–2014. Detailed information about mortality data is reported elsewhere [12]. A specific death code for MPM was not available until 2003, when the tenth revision of the International Classification of Diseases was implemented in Italy (MPM ICD codes: C38.4, C45.0, C45.9). Thus, the annual number of MPM deaths in the period 1970–2002 was estimated by applying a correction factor to the number of deaths for pleural cancers (ICD codes: VIII revision: 163.0; IX revision: 163.0–163.9) recorded in each year, as proposed by Ferrante and colleagues [24].

We computed annual asbestos consumption using data on domestic production, importation, and exportation as described in the paper by Marinaccio and colleagues [22]. We used the demographic statistics of the Italian population for the period 1970–2014 and projections for the period 2015–2039, as estimated by ISTAT (http://demo.istat.it/index_e.htm, last accessed on 30 October 2020), to calculate per capita average consumption of asbestos over the study period. To evaluate the lifetime exposure to asbestos of the subjects, we first cross-classified them by period (from 1970 to 2014) and age (from 0 to 99 years). Then, for each of the 4545 yearly combinations (45 observed periods and 101 age classes), we calculated the related exposure history with a pre-defined lag period, setting lag 0 equal to the exposure that occurred during the last year. Therefore, assuming a maximum lag of 60 years, we generated a 4545 × 60 matrix of exposure histories (E) that was used to define temporal dependencies using the model presented in the next section.

Reliable figures on asbestos consumption in Italy are not available before 1946. We therefore assumed that asbestos consumption in earlier periods was equal to that estimated for 1946. This assumption was then modified in a sensitivity analysis. Similarly, official figures of asbestos importation and production dropped to zero after the 1992 national ban. However, it is reasonable to expect that some level of exposure continued to occur after that year, due to consumption of previously stocked material, use of tools containing asbestos, and the presence of large amounts of asbestos-cement in buildings. We therefore explored different assumptions regarding post-ban asbestos exposure, corresponding to 5%, 10%, 20%, and 30% of the last consumption data recorded in 1992. Finally, even if theoretically asbestos exposure can occur at any age, occupational exposures experienced during the working age were arguably the most relevant. We thus explored different age ranges during which asbestos exposure likely occurred (15–65, 15–70, 20–65, and 20–70 years), and we considered exposed in each yearly period only the subjects in these age ranges. For the purpose of the present study, we explored all the combinations of the aforementioned assumptions and we selected the best fitting model following a model selection procedure described in the next section.

### 2.2. Statistical Models

We modelled the association between past asbestos exposure and MPM mortality rates using a quasi-Poisson generalized linear model with age/year-specific MPM deaths as the outcome and the corresponding log-transformed population as an offset. The model included a term for modelling the cumulative risk of MPM mortality based on the full exposure history within lag 0–60. This term was defined using distributed lag non-linear models (DLNMs), a flexible methodology that can specify complex exposure–lag–response associations where the risks vary depending on the intensity and timing of exposure [23]. Specifically, the DLNM is described by a bi-dimensional cross-basis function:

(1) $$s\left(x,t\right)\approx \overset{60}{\underset{l=0}{\sum }}f\left(x_{t-l}\right)w\left(l\right)$$

parameterized by a tensor-product combination of an exposure–response function *f(x_t−l_)* and a lag–response function *w(l)*. Alternative DLNMs can be defined through independent choices for these two functions, for instance, simple linear/constant terms or more complex non-linear shapes through spline transformations. Algebraic details and definitions are provided elsewhere [23]. The quasi-Poisson GLM was fitted to the observed mortality data for the period 1970–2014 to estimate the coefficients of the cross-basis function, and then used to forecast the MPM deaths in 2015–2039.

### 2.3. Model Selection and Sensitivity Analysis

We used natural splines as basis functions for defining the parametric form of both *w(l)* and *f(x_t−l_)*. The default intercept in *w(l)* was excluded from the models under the assumption that the exposure to asbestos in the last year was not associated with an increased risk of death for MPM. However, different choices regarding the specification of the spline functions for *w(l)* and *f(x_t−l_)*, such as number and location of knots, in addition to alternative assumptions about the age range and post-1992 exposure (see the section Data), resulted in a considerably large number of models. To deal with the issue of selecting the optimal model for predicting the future numbers of MPM and describing the exposure–lag–response association, we validated all models taking advantage of an out-of-sample (OOS) approach [25]. Given the complexity of the studied phenomena, we avoid a cross-validation step in favor of a OOS one. Empirical evidence from Cerqueira et al. [25] indeed confirm how OOS yields more reliable estimates in presence of time series originated from complex structures.

**Figure 1:**
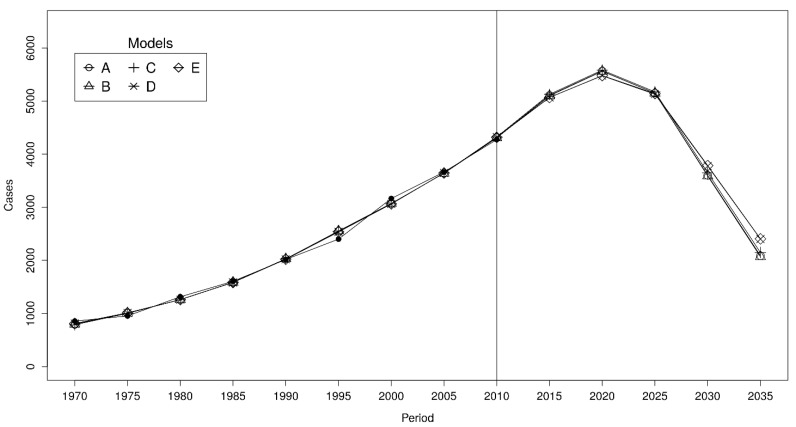
Comparisons of predictions based on the five best performing models. Model A assumes an age range for exposure equal to 20–70 years and a post-ban asbestos exposure equal to 10% of that estimated in 1992. Models B, C, D, and E assume (20–70 years; 5%), (20–70 years; 20%), (20–65; 10%), and (20–65; 5%), respectively. Black dots indicate observed cases.

We split our observed time series of MPM into two parts: an initial fit period (Training Dataset, from 1970 to 2004) in which the DLMN was trained, and a testing period (Test Dataset, from 2005 to 2014) held out for assessing the model predictions in term of mean squared error (MSE) [26]. Then, among all the possible models, we selected the one with the minimum MSE computed on the Test Dataset. To check whether the results change substantially using models that had a slightly higher MSE than the selected model, we compared the forecasts of the five best performing models (Figure 1).

Finally, we carried out two sensitivity analyses to evaluate the effect of our assumptions on the prediction of future MPM cases. First, we evaluated whether some of the excluded models forecasted a substantially different trend of MPM deaths compared to the selected one. For this reason, we compared the predictions of all models that had a fit to the data similar to that of the best model (i.e., less than a 2-point difference in MSE). Second, we modified the key assumptions of the selected model, one by one, to explore which had a higher influence on predictions. In particular, we assessed the following assumptions: different exposure age; linear increment of asbestos exposure from 1881 to 1945, rather than a constant value; and restriction of the analysis to individuals aged between 50 and 89 years old (see Figure S2).

## 3. Results

### 3.1. Descriptive Statistics

**Figure 2:**
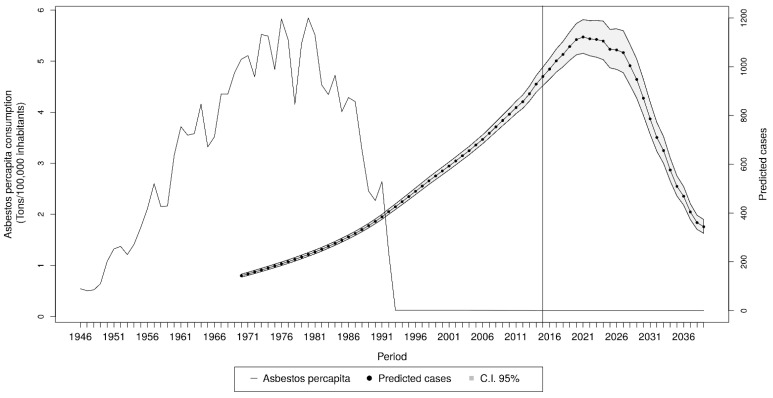
Fitted and predicted (after 2014) MPM cases with related 95% CI. To the left, asbestos per capita consumption in the period 1946–1992 in Italy.

Since the end of World War II, asbestos per capita consumption increased exponentially in Italy until the late 1970s, reaching its peak at nearly 6 tons/100,000 inhabitants. Thereafter, the consumption sharply decreased, dropping to zero after 1992, when asbestos production and consumption was banned in the whole country (Figure 2). A constant increase in MPM deaths (from 152 to 960 cases per year) was reported over the period 1970–2014.

### 3.2. Estimation of the Exposure–Lag–Response Association through DLNMs

A comparison of all implemented models with a different set of assumptions is reported in Supplementary Materials (Table S1), sorted by MSE. For the sake of clarity, we listed only models already optimized with respect to the number and the position of knots. The best performing model assumed an age range for asbestos exposure between 20 and 70 years and a post-ban exposure equivalent to 10% of that estimated in 1992. The performance of the selected model is displayed in Figure S1.

**Figure 3:**
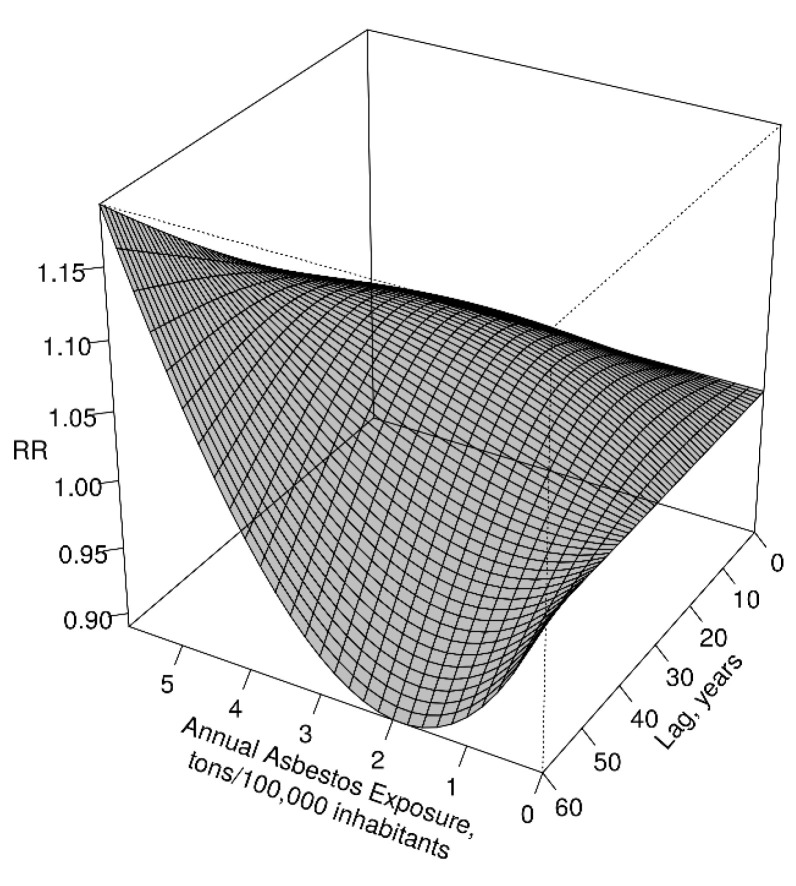
Exposure–lag–response association between the number of MPM deaths and the related past asbestos exposure (tons/100,000 inhabitants). Tridimensional exposure–lag surface.

**Figure 4:**
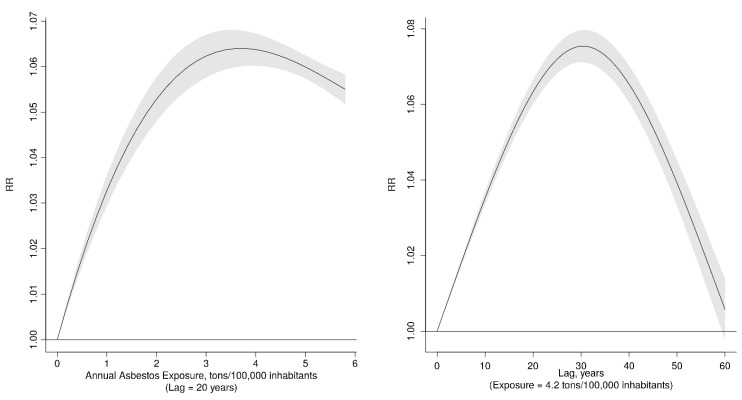
Exposure–response curve at lag 20 years (**left**) and lag–response curve for annual asbestos exposure of 4.2 tons/100,000 inhabitants (**right**), with 95% CI.

Figure 3 displays the full exposure–lag–response throughout a bi-dimensional representation of the predicted risk over a grid of exposures and lags, while Figure 4 shows the risk of MPM for specific values of exposure and lag time. The exposure–response curve at a lag of 20 years, in the first panel of Figure 4, shows that the Relative Risk (RR) of MPM increased almost linearly for lower levels of exposure but flattened at higher levels. The shape of the lag–response (latency) curve for an exposure of 4.2 tons for 100,000 inhabitants, plotted in the second panel of Figure 4, shows interesting temporal patterns. The lag-specific RR grew until 30 years since exposure and decreased thereafter, suggesting that the most relevant contributions to the risk come from exposures that occurred 20–40 years before death, but the RR keep increasing almost linearly for 30 years after the exposure.

### 3.3. Predicted Mortality

Forecasts for the years 2015–2039 are reported in Figure 2. According to our model, the peak of MPM deaths is expected to occur in 2021, with 1122 (95% IC 1055–1193) expected cases. Thereafter, we forecasted a constant decrease in mortality, down to 344 (95% CI 317–373) annual MPM deaths in 2039, about 30% of that expected during the peak. Results suggest that the predictions did not vary substantially after changing our basic assumptions. In particular, predictions were quite robust with respect to the assumption on the amount of asbestos exposure that occurred after the national asbestos ban (5%, 10%, or 20% of 1992 exposure), or including a linear increasing trend for the period 1881–1946.

**Figure 5:**
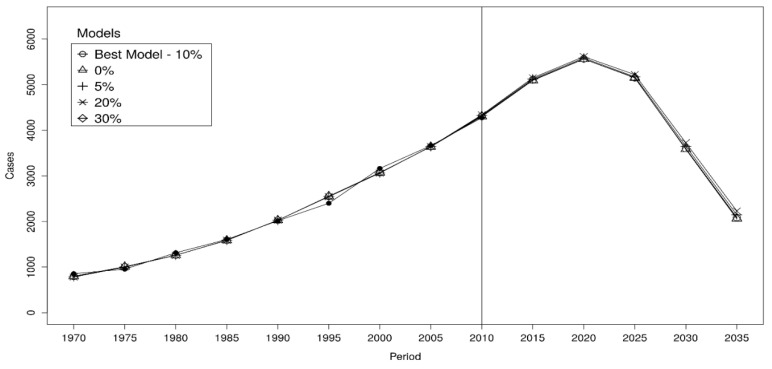
Predictions based on different assumption on asbestos exposure after 1992. Black dots indicate observed cases.

Finally, in Figure 5, we report a wider sensitivity analysis, varying some key assumptions of the main model, one at a time. In particular, we evaluated the effect of (i) using a linear increment of asbestos exposure from 1881 to 1945 instead of a constant value, (ii) varying exposure age ranges, and (iii) restricting the analysis to individuals aged between 50 and 89 years old. As before, alternative assumptions did not impact much on our predictions, with all models converging towards a significant decrease in MPM deaths after 2021 and similar expected mortality in 2039. Further research can also investigate and compare bootstrap and classical standard error estimates of model parameters.

## 4. Discussion

In the present study, we used a novel approach to predict the number of future cases of malignant pleural mesothelioma (MPM), in terms of past asbestos consumption. Our results suggest that we are finally approaching the peak of the number of MPM deaths in Italy.

Previous studies suggest that the pattern of incidence/mortality for MPM follows the pattern of asbestos use with a lag of about 40 years. Lin et al. [27] analyzed the relation between national asbestos consumption and asbestos-related disease mortality in 30 countries worldwide, observing a 1.81 and 1.33-fold increase in mesothelioma death rates in men and women, respectively, for each kg of asbestos used (per capita) 40 years before. The relation of asbestos national use and mesothelioma occurrence was also investigated in specific countries, including Brazil [19], Italy [22], Spain [21], the Netherlands [28], the UK [14], and the USA [29], suggesting similar time patterns in asbestos consumption and mesothelioma occurrence. An original contribution of our study is that we flexibly modelled the contribution of asbestos exposure to MPM risk for the whole latency period, rather than simply estimating the lag time at which the association was stronger. Our results suggest that all exposures occurring between 20–40 years before the development of MPM are associated with an increase in risk and that the risk increased also for shorter lag time after exposure. The risk was the highest for exposures that occurred 30 years before and declined thereafter. This result is in contrast with the classic assumption that the MPM risk increases indefinitely with time since asbestos exposure (HEI, 1991). On the other side, our results are consistent with more recent studies showing that MPM risk levels off many years after the first asbestos exposure, thus entailing that remote exposures could have a lesser role in the development of MPM than previously assumed [30,31].

Even if the analysis used official national figures, patterns of asbestos use and of mesothelioma occurrence are not uniform in the country, being related to industrial activities, in particular naval construction, asbestos cement, automotive, railways carriages, constructions, chemical, textile, and other industries, more often located in the north of Italy or in areas of special interest, such as harbors and coastal areas [32]. Albeit a regional analysis of data was of interest, the available information on asbestos use could not be reliably considered at the regional level. Limited to the area of Casale Monferrato, where the largest Italian asbestos cement factory has been operating, a time trend analysis was conducted by Furlan and Mortarino [33]. They estimated the extent of mesothelioma epidemic in the area considering the amount of asbestos used in the local asbestos cement factory and forecasted the epidemic to last until 2028–2031. Little is known about the extent of asbestos exposure after the national ban was implemented in 1992. On one side, the production and use of asbestos effectively ceased, completing a process started a few years earlier [9,22], translating into a sharp reduction in active and bystander exposure. On the other side, a large amount of asbestos containing material remained in place after the ban. The asbestos insulation was cleared earlier while the asbestos cement materials in place are still being removed and the process is far from being completed [34]. In this respect, we applied different estimates of exposure in sensitivity analyses, observing limited changes in the predicted number of MPM.

In previous studies, we used an age–period–cohort (APC) approach to investigate pleural and peritoneal mesothelioma mortality trends in Italy. According to these analyses, the MPM peak has not yet been reached in Italy, as opposed to peritoneal mesothelioma [12,35]. In particular, APC models predict the peak of MPM cases in men to be reached in the period 2020–2024 (about 5200 cases), followed by a slow decrease, with 19,500 MPM cases expected to occur in Italy over the next 20 years (2020–2039) [12]. Results of the present analyses are consistent with the indications from these previous studies regarding the time of the peak, but the following decrease is expected to be steeper. This is probably because models based on asbestos consumption capture the effect of the implementation of the 1992 national asbestos ban more accurately than APC analysis [12]. Despite the fall in the number of MPM predicted in the next years, it is noteworthy that more than 300 MPM cases are still expected to occur in 2039, a number similar to those observed during the late 1980s. This suggests that MPM epidemic in Italy is far from being over.

The economic cost of each case of MPM is relevant. A recent Italian study highlighted that medical care, insurance and compensation, and productivity loss could result in about EUR 230,000–250,000 per each MPM case [36]. Thus, considering a forecast of about 15.500 MPM deaths within the period 2020–2039 only among men, the total amount could be estimated in about EUR 3,565,000,000 and 3,875,000,000. No economic burden estimations were available for our country regarding asbestos-related diseases (ARDs) other than MPM, but it is reasonable that this economic burden would significantly increase also taking into consideration lung cancers due to asbestos exposure [37] and other ARDs. Moreover, predictions of MPM mortality is a precious tool for public health authorities and physicians to program practices and interventions. Our model could represent a reference to perform MPM predictions analyses in countries with a current use of asbestos, and this could support a costs-benefits evaluation of the potential asbestos ban, as recommended by several independent agencies and international public health authorities.

Our analyses are based on mortality records from MPM, which offer the advantage of a longer period of observation compared to the registration of incident cases of MPM. National mortality data on pleural cancer deaths were reported to underestimate the actual number of mesotheliomas, due to misdiagnosis, non-diagnosis, or lack of reporting [2,38]. However, the pattern of mesothelioma death rates observed in our data is similar to that estimated by other data sources, such as the GBD database [3,12]. We used mortality data according to the availability of nationwide figures for the entire observed period of this data source. Furthermore, according to the poor survival period for MPM cases, incidence and mortality are strictly correlated, and previous studies [39] showed that incidence and mortality rates for mesothelioma in Italy are remarkably similar.

Our study has some limitations. We based our forecasts only on male MPM mortality, and thus these could not be entirely referred to both genders due to differences in circumstances of exposure, with a higher number of cases among women who have had environmental or extra-occupational asbestos exposures (28.3% for women vs. 4.5% for men) [40]. Previous studies suggest that asbestos consumption is mainly a proxy for occupational asbestos exposure, which is the most important type of exposure in men. For this reason, previous studies that provided forecasts based on historical asbestos exposure are typically restricted to men [14,22], and we followed this approach, as well. Moreover, we adopted asbestos national consumptions as proxy of asbestos exposure at national level. It is unquestionable that asbestos exposure is a complex metric that involves the number of exposed workers, the type of asbestos fibers, the economic sectors and job involved and the implementation of preventive measures. The pure distribution of asbestos consumption by year at national level cannot consider these topics. Nevertheless, it was well documented that the asbestos consumption curve (in the past) is a good predictor of MPM mortality (or incidence) after an adequate lag period, and exercises of prediction of MPM epidemic curve based on this metric have been just performed successfully in many countries [22,41]. Furthermore, our assumption that asbestos consumption in earlier periods was equal to that estimated for 1946 has to be considered a limitation of the study, according to the plausibility of an increasing trend between 1920–1946, although including a linear increasing trend for the period 1881–1946 does not significantly affect model forecasts (Figure S2, model A4). Finally, our predictions could be limited by improvements of diagnostic tools and therapeutic advancements. At present, MPM has poor survival and limited therapeutic options [42], but clinical improvements could enhance both diagnosis [43] and therapy [44], allowing a reduction in future mortality rates and thus modifying future number of MPM deaths.

## 5. Conclusions

According to an analysis based on historical asbestos consumption, the epidemic of MPM in Italy will reach its peak in 2021 with a predicted number of 1122 (95% CI 1055–1093) cases among men, followed by a steep decrease to 344 (95% CI 317–373) annual MPM deaths in 2039, about a third of that expected during the peak. Our results also show that the association between temporal trends of MPM and time since asbestos exposure is not monotonic, suggesting a lesser role of remote exposures in the development of MPM than previously assumed.

## Data Availability

The demographic statistics of the Italian population for the period 1970–2014 and projections for the period 2015–2039 could be found at ISTAT website (http://demo.istat.it/index_e.htm, last accessed on 30 October 2020).

## Supplementary Materials

The following are available online at https://www.mdpi.com/article/10.3390/cancers13133338/s1. Figure S1: Performance of the main model on the test dataset. Figure S2: Models predictions under different assumptions. A is the best model with an exposure age (EA) equal to 20–70 years old, an asbestos exposure (AE) after 1992 equal to 10%, a maximum lag of 60 years, an AE before 1946 equal to the AE observed in 1946, analysis restricted to cases aged 25–89 years old. A1: equal to A, except for the EA assumed to be 15–70 years old. A2: equal to A, except for the AE assumed to be 20–65 years old. A3: equal to A, except for the restriction of analysis to cases aged 50–89 years old. A4: equal to A, except for AE before 1946, assumed to linearly increase from 1881 to 1946. Black dots indicate the observed cases. Table S1: Comparison between different models.
