# Supplementary material for: Effect of Asbestos Consumption on Malignant Pleural Mesothelioma in Italy: Forecasts of Mortality up to 2040
Source: Cancers (Basel). 2021 Jul 3;13(13):3338. doi: 10.3390/cancers13133338 (PMC8267929; doi:10.3390/cancers13133338)
Supplement: Supplementary file 1 [file cancers-13-03338-s001.zip › cancers-1284424-supplementary.pdf]

cancers

MDPI

# Supplementary Materials: Effect of Asbestos Consumption on Malignant Pleural Mesothelioma in Italy: Forecasts of Mortality up to 2040

**Enrico Oddone, Jordy Bollon, Consuelo Rubina Nava, Dario Consonni, Alessandro Marinaccio [6], Corrado Magnani, Antonio Gasparrini and Francesco Barone-Adesi**

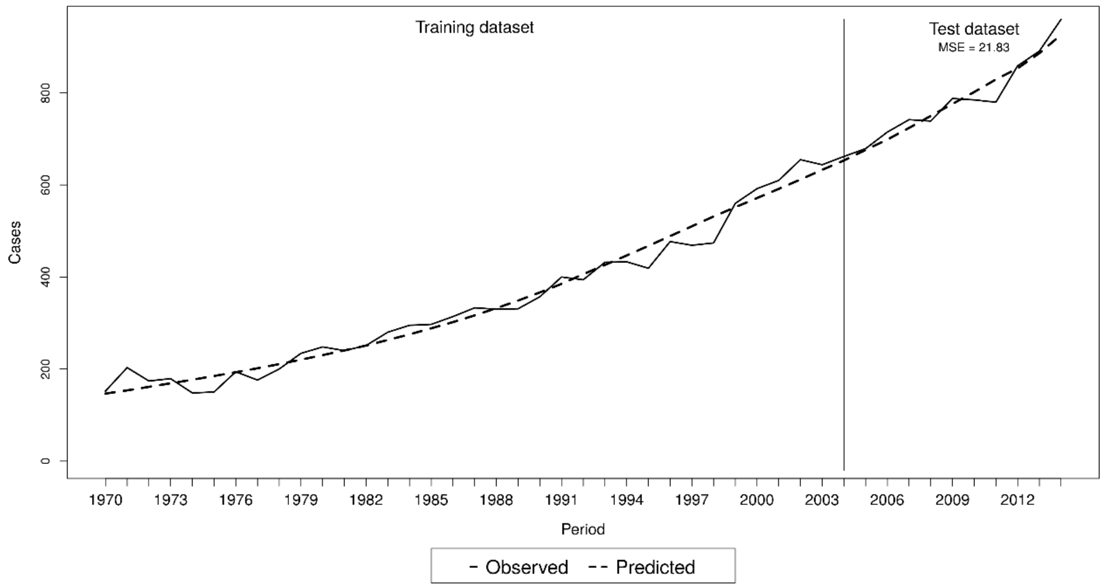


**Figure S1.** Performance of the main model on the test dataset.

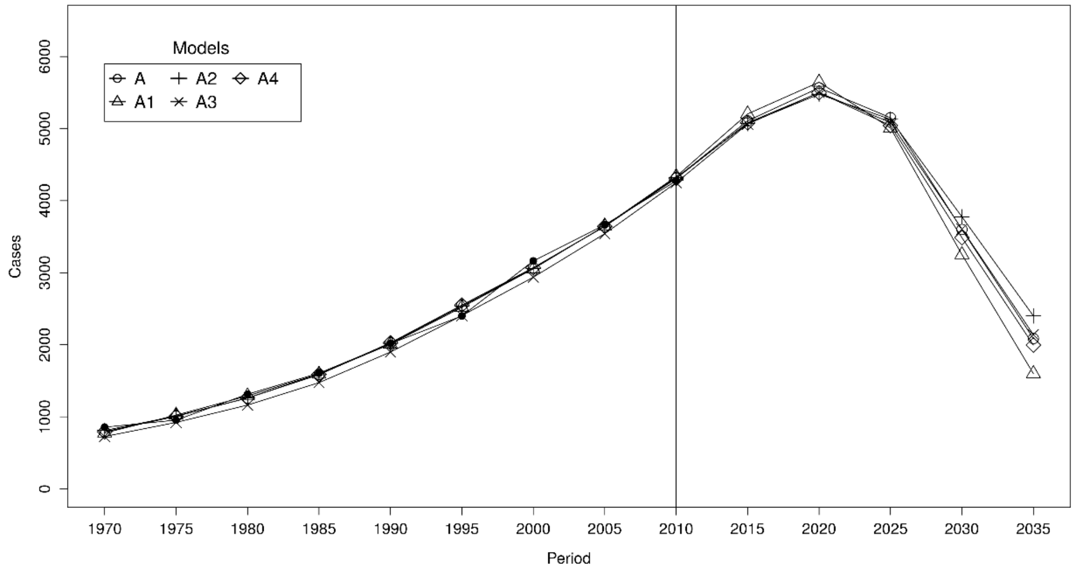


**Figure S2.** Models predictions under different assumptions. A is the best model with an exposure age (EA) equal to 20–70 years old, an asbestos exposure (AE) after 1992 equal to 10%, a maximum lag of 60 years, an AE before 1946 equal to the AE observed in 1946, analysis restricted to cases aged 25–89 years old. A1: equal to A, except for the EA assumed to be 15–

70 years old. A2: equal to A, except for the AE assumed to be 20–65 years old. A3: equal to A, except for the restriction of analysis to cases aged 50–89 years old. A4: equal to A, except for AE before 1946, assumed to linearly increase from 1881 to 1946. Black dots indicate the observed cases.

**Table S1.** Comparison between differnt models.

| Models | Age Range for Asbestos Exposure | Post–ban Exposure, Expressed as Percentage of the Exposure in 1992 | Test MSE |
|---|---|---|---|
| A | 20–70 | 10% | 21.83 |
| B | 20–70 | 5% | 22.53 |
| C | 20–70 | 20% | 22.76 |
| D | 20–65 | 10% | 22.85 |
| E | 20–65 | 5% | 22.98 |
| F | 20–65 | 20% | 23.39 |
| G | 20–65 | 30% | 25.24 |
| H | 20–70 | 30% | 26.91 |
| I | 15–70 | 5% | 30.41 |
| J | 15–70 | 10% | 36.84 |
| K | 15–65 | 10% | 37.23 |
| L | 15–65 | 5% | 41.63 |
| M | 15–65 | 20% | 43.25 |
| M | 15–70 | 30% | 44.68 |
| N | 15–70 | 20% | 51.55 |
| O | 15–65 | 30% | 66.04 |
